# DeRAS I – deutsche Situation der robotisch assistierten Chirurgie – eine Online-Survey-Studie

**DOI:** 10.1007/s00104-021-01404-x

**Published:** 2021-06-25

**Authors:** C. M. Krüger, O. Rückbeil, U. Sebestyen, T. Schlick, J. Kürbis, H. Riediger

**Affiliations:** 1 Abteilung Chirurgie/Zentrum für Robotik, Immanuel Klinikum Rüdersdorf, Seebad 82/83, 15562 Rüdersdorf b. Berlin, Deutschland; 2 SurgiData UG, Mahlow, Deutschland; 3 Department für Chirurgie, Vivantes Humboldt Klinikum, Berlin, Deutschland

**Keywords:** Robotik, Innovation, Digitalisierung in der Medizin, Implementierung, Umfrage, Robotics, Innovation, Digitalization in medicine, Implementation, Survey

## Abstract

**Hintergrund:**

Die robotische Assistenz hat sich in der Chirurgie etabliert, ist aber noch kein Standard. Der aktuelle Stand der klinischen Verbreitung in Deutschland ist weiter unklar. Industrieunabhängige Quellen sind rar.

**Ziel der Arbeit:**

Ziel dieser Umfrage ist es, den aktuellen Stand der robotisch assistierten Chirurgie (RAS) fachübergreifend in Deutschland im Zeitraum von 2014 bis 2018 zu untersuchen.

**Materialien und Methoden:**

Mit einer Internetrecherche wurden Krankenhäuser (KH) und Fachabteilungen (FA) mit Zugang zur RAS identifiziert. Die FA wurden aufgefordert, ihre Daten aus den Jahren 2014 bis 2018 zu teilen. Neben klinischen Daten wurden Daten zu Nutzung, Implementierung, Training und Finanzierung abgefragt.

**Ergebnisse:**

Am 31.12.2018 wurde die RAS an 121 KH in Deutschland angeboten. 383 FA mit Zugang zur RAS wurden identifiziert. 26 % (*n* = 98) der FA haben geantwortet. Im Mittel verfügte jede FA über zwei Konsolenchirurgen. 10 % der KH verfügten über mehr als 1 RAS-System. 100 % der erfassten RAS-Systeme stammten von der Firma Intuitive Surgical Inc., CA, USA. Die RAS wurde zu 65 % in der Urologie implementiert, zu 12 % in der Viszeralchirurgie (VC). 21 % der Programme erfolgten interdisziplinär und 4 % multidisziplinär (> 3). 83 % der Systeme wurden gekauft, 17 % anderweitig finanziert. Bei den Operationsmehrkosten gaben 74 % der Kliniken an, diese selbst zu tragen. 14 % wählten eine Umlage. Seit 2014 steigerten sich die Eingriffe um den Faktor 4 auf ca. 8000. Der Anteil der VC steigerte sich um das Fünffache seit 2016.

**Schlussfolgerung:**

Die RAS erlebte in Deutschland bis 2018 ein starkes Wachstum. Das Eingriffsspektrum entspricht dem der Laparoskopie. Bei aktuell fehlender Kostenerstattung für den technischen Mehraufwand, wird die RAS überwiegend im mittel- und hochkomplexen Bereich eingesetzt. Der Online-Survey ist eine gute Methode, ohne hohen administrativen Aufwand unabhängige Daten zu erheben.

## Abstract (en)

**Background:**

Robotic assistance has become established in surgery but is not yet a standard procedure. The current status of clinical dissemination in Germany remains unclear. Industry independent sources are scarce.

**Aim of the work:**

The aim of this survey was to investigate the current status of robotic-assisted surgery (RAS) across specialties in Germany from 2014 to 2018.

**Material and methods:**

An internet search was used to identify hospitals and departments (DP) with access to RAS. The DPs were asked to share their data from 2014–2018. In addition to clinical data, data on utilization, implementation, training, and funding were requested.

**Results:**

As of 31 December 2018 RAS was offered at 121 hospitals in Germany, 383 DPs with access to RAS were identified and 26% (*n* = 98) of DPs responded. On average each DP had two consultant surgeons, 10% of DPs had more than one RAS system and 100% of the RAS systems recorded were from Intuitive Surgical Inc., CA, USA. RAS was implemented in 65% in urology and in 12% in visceral surgery (VS). 21% of programs were interdisciplinary and 4% multidisciplinary (> 3). 83% of systems were purchased and 17% otherwise funded. For additional operating room costs, 74% of hospitals reported paying for them themselves. 14% chose pay as you go. Since 2014, procedures increased by a factor of 4 to approximately 8000. The proportion of VS increased by a factor of 5 since 2016.

**Conclusion:**

RAS in Germany experienced strong growth through 2018. The range of procedures is similar to that of laparoscopy. With a current lack of reimbursement for the additional technical effort, RAS is predominantly used in the medium and high complexity range. The online survey is a good method to collect independent data without high administrative effort.

## Hintergrund

Die minimal-invasive Chirurgie (MIC) hat sich in den vergangenen 20 Jahren interdisziplinär etabliert. Beginnend mit Eingriffen niedriger Komplexität hat sie in den vergangenen 10 Jahren bei komplexen Indikationen, von kolorektaler Chirurgie, über die Magen- und Pankreaschirurgie bis hin zur Lungen- und Ösophaguschirurgie, ihren Platz eingenommen. Neben etablierten Standards liegen auch zahlreiche Untersuchungen zum medizinischen Mehrwert vor. In Teilbereichen ist auch in randomisierten Studien ein Vorteil belegt.

Wie sich die aktuelle Situation in Deutschland derzeit entwickelt, ist aus unabhängigen Quellen nur schwer zu ergründen – um dies zu klären, haben wir eine Survey-Umfrage über den Zeitraum 2014 bis 2018 durchgeführt.

Die robotisch assistierte Chirurgie (RAS) ist eine technische Weiterentwicklung der MIC. Im Gegensatz zum industriellen Einsatz von Fertigungsrobotern, deren Hauptcharakteristik, neben Präzision und Stückzahl pro Zeiteinheit, das programmierte selbstständige Arbeiten ist, ist in der RAS auch weiterhin die Hand des Chirurgen das bestimmende Moment. Die RAS bietet eine technische Unterstützung des Chirurgen. Merkmale sind hochauflösende 3‑D-Visualisierung, verbesserte Präzision, Tremorbereinigung und die Unterstützung komplexer intrakorporaler Bewegungsabläufe durch die Bereitstellung von bis zu 7 Freiheitsgraden beim Instrumentarium. Hierdurch werden komplexe Bewegungen, wie das zirkuläre Präparieren dreidimensionaler Strukturen und die robotische Nahtrekonstruktion, in technisch signifikanter Erleichterung am Patienten erbracht. Die aktuell am Medizinmarkt überwiegend vertretenen RAS-Systeme (daVinci-System, Intuitive Surgical Inc., CA, USA) statten den Operateur mit bis zu drei Instrumenten und einem 3‑D-HD-Kamerasystem unter eigenständiger Kontrolle aus.

Mit Markteinführung hat sich die RAS bis heute am stärksten in der Urologie (URO), insbesondere in der Prostatachirurgie, etabliert. Eine Übersicht mit Fokus auf die Viszeralchirurgie (VC) wurde 2016, damals mit 23 aktiven Zentren, von Kissler et al. publiziert [7].

Unser Anspruch war es, eine einfache Teilnahme über einen Online-Survey zu ermöglichen. Das Bearbeiten des Surveys sollte unter 10 min beanspruchen.

Kriterien, die durch die Erhebung des Surveys [9] herausgearbeitet werden sollten, wurden wie folgt definiert:

- Durchdringung der robotisch assistierten Chirurgie in der Krankenhauslandschaft in Deutschland in einem 4‑Jahres-Zeitraum,
- Erkenntnisse zum Operationsspektrum in den einzelnen Fachgebieten,
- Erkenntnisse über die eingesetzte RAS-Technologie,
- Analyse der strukturellen und ökonomischen Implementierung der RAS.

## Material und Methodik

Im Rahmen einer Online-Befragung wurden Daten zum Einsatz der RAS in Deutschland von 2014 bis 2018 erhoben. Zur Identifikation möglicher Kliniken, die über eine RAS verfügen, wurde eine systematische, internetbasierte Suche erstellt. Ausgehend von einer im Internet verfügbaren Liste aller deutschen RAS-Prostatazentren [13] wurden die Trägerkliniken identifiziert (Suche I). Anschließend wurden die weiteren vorhandenen Fachabteilungen (FA) für Allgemein- und VC, Gynäkologie (GYN), Herzchirurgie (HC), Thoraxchirurgie (THX) und Hals-Nasen-Ohren-Heilkunde (HNO) evaluiert, in denen die RAS eine klinische Zulassung besitzt (Suche II). Die Internetpräsenzen dieser Fachabteilungen wurden nach wiederkehrenden Schlagworten durchsucht, mit denen die Robotik als Teil des Leistungsspektrums besonders hervorgehoben wurde. Im dritten Schritt erfolgte anhand der gewonnen Schlagworte aus der Suche I und II die freie Internetsuche nach Kliniken, die eine klinische Internetpräsenz ihrer RAS anbieten. Unabhängig von den gefundenen Klinik und den dargestellten Fachabteilung wurden ebenso diese Kliniken auf die möglichen genannten Fachbereiche hin evaluiert und alle Kontaktdaten der Datenbank hinzugefügt (Suche III). Kliniken, die Robotik nicht als Teil ihres Leistungsspektrums auf der Webseite dargestellt hatten, wurden demnach nicht mit einbezogen.

Der Onlinefragebogen (https://www.surveymonkey.de/r/DERAS2018) war für 2 Monaten verfügbar. Am 15.05.2019 endete die Datenerhebung. Um eine hohe Akzeptanz zu erreichen, haben wir eine maximale Bearbeitungszeit von 10 min angestrebt. Innerhalb der Survey-Befragung wurden allgemeine Daten zur FA, zu RAS-spezifischen Daten, zu Fallspektrum, -menge, den Eingriffsarten, Implementierung, Training und Finanzierung erhoben. Ab dem 15.05.2019 war die Datenerhebung beendet.

## Ergebnisse

Anhand der Suchkriterien wurden im Studienzeitraum bis 2018 insgesamt 383 FA an 121 Krankenhäusern (KH) ermittelt. Die FA verteilten sich auf 103 URO, 106 VC, 95 GYN, 34 THX, 30 HNO und 15 HC.

Von den angeschriebenen FA antworteten anteilig: URO 27 %, VC 49 %, GYN 15 %, THX 0 %, HNO 10 % und HC 7 %. 98 FA haben geantwortet. Dies entspricht einer Rücklaufquote von 26 %. Die durchschnittliche Dauer der Survey-Bearbeitung lag bei 7 min 38 s (Ziel < 10 min). Die 98 bearbeiteten Fragebögen verteilten sich wie folgt auf die Fachabteilungen: 28/URO (29 %), 52/VC (53 %), 14/GYN (14 %), 0/0 THX (0 %), 3/HNO (3 %) und 1/HC (1 %).

Die Abteilungsgröße lag im Median bei 53 (10–100) Betten. Durchschnittlich waren 2 Fachärzte (1–9) als RAS-Operateure ausgebildet. Die Facharztqualifikation wurde im Median 10 Jahre vor dem Studienzeitraum erreicht (2004; 1983–2016).

Zur RAS wurden ausschließlich daVinci-Systeme der Firma Intuitive Surgical Inc., CA, USA eingesetzt. 21/98 Fachabteilungen machten keine Angaben zum Hersteller. In 71 Fachabteilungen wurde ein RAS-System und in 8 Fachabteilungen sogar zwei RAS-Systeme eingesetzt.

Eingesetzt wurden 32-mal das daVinci-Si-System (3. Generation), 33-mal das daVinci-Xi-System (4. Generation), 19-mal das daVinci-X-System (4. Generation ab 2017) und 2‑mal das daVinci-Single-Port-System als Erweiterung der 3. und 4. Generation. Zweimal wurde die Gerätekombination daVinci Si+Xi, einmal daVinci Si+X, dreimal daVinxi Xi+X und zweimal die Kombination daVinci X+ Single-Port angegeben. Somit waren in den 98 Fachabteilungen insgesamt 86 daVinci-Systeme in Betrieb.

Auf die Frage nach der Fachabteilung, die erstmalig die RAS an der Klinik etabliert hatte, haben 82 von 98 (80 %) Abteilungen geantwortet. Hierbei ergab sich, dass bei 65 % der Kliniken (*n* = 53) die URO das klinische Programm alleine gestartet hatte. In 12 % startete die VC (*n* = 10) und einmal (1 %) die GYN. In 21 % wurde der Programmstart interdisziplinär getätigt (*n* = 17). Die Kombinationen waren wie folgt vertreten: URO/VC (*n* = 6), URO/GYN (*n* = 7) und URO/THX (*n* = 1). In 3 Fällen wurde der Programmstart multidisziplinär durchgeführt: URO/THX/VC (*n* = 2), URO/VC/GYN/HNO/HC (*n* = 1).

Ein weiterer Themenkomplex der Befragung richtete sich auf die Dauer der Programmimplementierung bezogen auf die Technik und das Training des Personals. Hierbei wurden die Kliniken nach der durchschnittlichen Dauer zwischen der Vertragsunterschrift mit dem Hersteller und dem ersten chirurgischen Eingriff, der Zeitspanne zwischen der Lieferung und Installation in der FA und dem ersten chirurgischen Eingriff sowie der Zeitspanne zwischen dem Beginn des chirurgischen Konsolentrainings und dem ersten chirurgischen Eingriff befragt.

Von den 98 rückgesendeten Fragebögen wurden 72 (73 %) wie folgt beantwortet:

- Unterschrift Vertrag bis erster chirurgischer Eingriff: 3,9 Monate,
- Lieferung/Installation des Systems bis erster chirurgischer Eingriff: 2,2 Monate,
- erstes chirurgisches Training bis erster chirurgischer Eingriff: 1,9 Monate.

Für alle drei Zeiträume lagen die genannten Mittelwerte in einer Spanne von 1 bis > 6 Monate.

**Figure 1:**
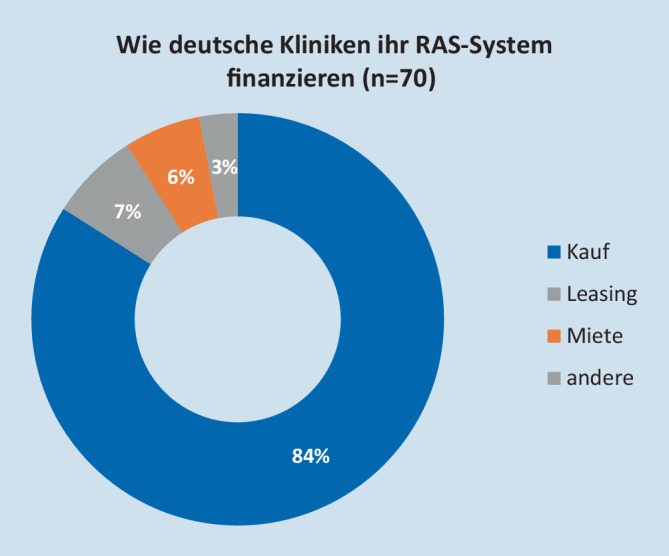

**Figure 2:**
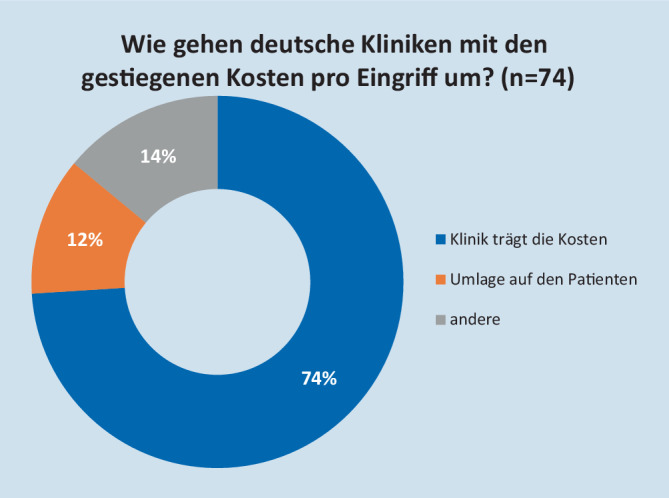

Die Investition in das RAS-System erfolgte zu 84 % durch Kauf, zu 7 % durch Leasing, zu 6 % durch Miete (Abb. 1). Die Kosten wurden in der Regel (74 %) aus dem Klinikbudget getragen. Immerhin 12 % gaben an, die Mehrkosten auf die Patienten weiterzugeben (Abb. 2). Wie die Umlage auf die Patienten erfolgte, wurde nicht benannt.

**Figure 3:**
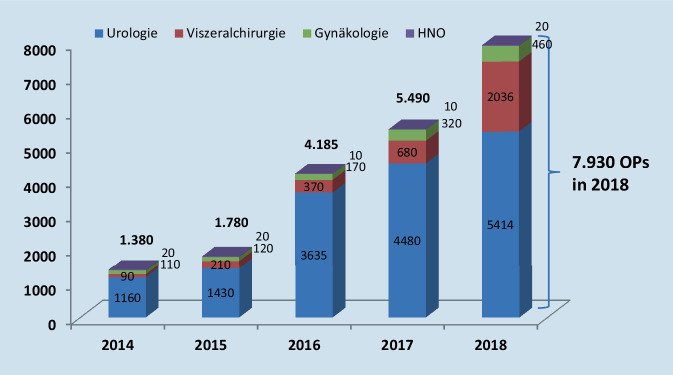

Zusammenfassend kann gesagt werden, dass der überwiegende Teil der Kliniken zum Zeitpunkt der Erfassung die Anschaffung durch Kauf getätigt und die fallbezogenen Mehrkosten im eigenen Sachkostenbudget getragen hat. Abb. 3 zeigt die Entwicklung der Eingriffe von 2014 bis 2018 nach Fachbereichen getrennt. Für das Jahr 2018 wurden detailliert nach Eingriffsspektrum 7930 Eingriffe durchgeführt. Davon entfielen auf die URO 5414, die VC 2036 und auf die GYN 427 Operationen. Für die URO entfielen erwartungsgemäß 59 % der Eingriffe auf die radikale Prostatektomie (*n* = 3180). 53 % der in der VC durchgeführten Operationen wurden durch das kolorektale Spektrum gestellt (*n* = 1074). Ein Großteil der in der VC durchgeführten Eingriffe fand bei benignen Indikationen statt.

In der GYN entfielen auf die Hysterektomie (*n* = 330) 77 % der Eingriffe.

## Diskussion

Die RAS nahm ihren Anfang in der HC [3, 15] zum Wechsel des Jahrtausends, konnte sich hier aber aufgrund der begrenzten Performance der frühen Systemgenerationen ([14]; S‑System, Intuitive Surgical Inc., CA USA) nicht etablieren. Einige Jahre später tritt sie ihren Siegeszug in der URO mit der Prostatektomie an [6, 12] und setzt diesen Trend in der GYN fort [4, 11]. In diesen Fachbereichen wird auf komplexe, onkologische Eingriffe fokussiert (Tab. 1 und 2).

**Table 1:** 

RAS-Prozeduren Urologie	Gesamtanzahl Eingriffe in allen Zentren
Prostatektomie	3180
Partielle Nephrektomie	758
Eingriffe am Urether	620
Pyeloplastie	315
Komplexe Blaseneingriffe	290
Totale Nephrektomie	250
Nierenlebendspende	1
Summe RAS-Eingriffe Urologie 2018	5414

*RAS* robotisch assistierte Chirurgie

**Table 2:** 

RAS-Prozeduren Gynäkologie	Gesamtanzahl Eingriffe in allen Zentren
Hysterektomie (benigne)	200
Hysterektomie (maligne)	130
Sakrokolpopexie	82
Myomektomie	15
Summe gyn. RAS-Eingriffe 2018	427

*RAS* robotisch assistierte Chirurgie

Die VC, beginnend mit der kolorektalen Chirurgie, trat erst ab 2012 bis 2014 in Europa in den Fokus des chirurgischen Interesses und hat sich zu dem breitesten Anwendungsgebiet entwickelt (Tab. 3; [7, 8]).

**Table 3:** 

RAS-Prozeduren Viszeral‑, Thoraxchirurgie	Gesamtanzahl Eingriffe in allen Zentren
Kolon (benigne)	422
Rektum	407
Kolon (maligne)	245
Lobektomie	198
Ösophagus	188
Zwerchfellbruch	143
Gallenblase	83
Magen	73
Thymektomie	59
Pankreas	54
Wedge-Resektion	53
Leber	41
Bauchwandbruch	29
Nebenniere	21
Leistenbruch	18
Schilddrüse	2
Summe RAS-Eingriffe Visz.-, Thor.-Chirurgie 2018	2036

*RAS* robotisch assistierte Chirurgie

Ein Hemmschuh für die RAS in Deutschland und den Ländern, die dem Prinzip der DRG-Finanzierung folgen, ist die fehlende Berücksichtigung im Erlössystem. Die Erstattung des technologischen Mehraufwandes durch die gesetzlichen Krankenkassen erfolgt nur für Leistungen des Gegenstandskataloges. Die RAS ist hier aktuell nicht enthalten. Erst seit 2016 enthält das DRG-System einen OPS-Code (5-987.0), der die RAS identifizierbar macht, aktuell aber ohne Erlösrelevanz.

Die Kostenträger erwarten wissenschaftliche Evidenz, bevor neue Technologien erstattet werden. Somit stehen die Kliniken vor der Entscheidung, in Technologien zu investieren, ohne sicher sein zu können, den Mehraufwand für Investition, Etablierung und Lernkurve erstattet zu bekommen. Dieses Dilemma ist seit Jahren Gegenstand von Fachdiskussionen um den zukünftigen Stellenwert der RAS in operativen Disziplinen. Dies kann sich auf die Akzeptanz in der Breite der Patientenversorgung als Hemmnis auswirken.

Unsere Befragung schloss explizit RAS-Systeme möglicher weiterer Hersteller mit ein.

Die vorliegende Untersuchung ergibt Daten zur Entwicklung der RAS im interdisziplinären Vergleich. Eine Rücklaufquote von 26 % der Fachabteilungen (98/383) bzw. 49 % der Kliniken (59/121) wurde in vergleichbaren Untersuchungen als repräsentativ beschrieben [1, 10, 16].

In den vergangenen Jahren hat das Thema RAS in Deutschland und der Welt sowohl auf wissenschaftlichen Tagungen als auch in der medialen Präsenz in einem Maß zugenommen, dass es mitunter schwerfällt, eine reale Einschätzung der Istsituation abzugeben. Die Frage nach dem medizinischen Mehrwert der RAS kann aktuell nicht einheitlich beantwortet werden. Selbst für das Gebiet mit den höchsten Fallzahlen, die Prostatachirurgie, liegen nach 15 Jahren nur marginal randomisierte Studien mit ausreichender Aussagekraft vor [2, 5].

Aufkommender Wettbewerb und zum Teil aggressives Marketing lassen in der fachlichen Diskussion die Idee aufkommen, eine konventionell minimal-invasive Operation sei eine Methode zweiter Wahl und ein offen chirurgisch erbrachter Eingriff sogar der Ausdruck chirurgischen Unvermögens.

Die Einstiegshürden für eine Klinik in die RAS sind hoch. Zu berücksichtigen sind Investitionen, Betriebskosten, prozedurale Kosten, Training, Mindestmengen bei onkologischen Eingriffen und die zu berücksichtigenden Qualitätsindikatoren. Die klinische Herausforderung ist es, Innovation und technischen Fortschritt ohne Qualitätsverlust im medizinischen Alltag umzusetzen. Wir konnten zeigen, dass > 60 % der Programme durch Urologen mit einer Prozedur, der Prostatektomie, begonnen wurden. Die VC bietet mehr Indikationen, was die Lernkurve und die Geschwindigkeit der klinischen Umsetzung verlängert. Der VC-Fokus liegt heute auf den komplexeren und erlösträchtigeren Eingriffen, wie z. B. der kolorektalen, Ösophagus- und Pankreaschirurgie. Benigne Indikationen und Eingriffe mit großer Häufigkeit, wie z. B. Cholezystektomien und Herniotomien, sind weniger adressiert. Konnten Kissler et al.[7] 2016 erstmals 23 VC-Kliniken mit Zugriff auf ein robotisches Operationssystem in Deutschland ausmachen, haben wir 106 VC mit möglichem Systemzugriff detektiert. Hiervon haben sich immerhin 49 % an der Umfrage beteiligt.

Die VC zeigt in unserer Untersuchung ab 2016 mit einer Verfünffachung den größten Zuwachs bei den Prozeduren. Mit 53 % stellt die kolorektale Chirurgie das größte Segment dar. Benigne Indikationen wurden häufiger robotisch operiert als maligne. Wir interpretieren dies als ein Herantasten an die Technologie im Sinne der derzeit stattfindenden Lernkurve. Bezogen auf die durchgeführten RAS-Operationen zeigen die Zahlen aus 2018, dass im Bereich der URO allein mit der Prostatektomie ein Anteil von > 50 % des jährlichen Gesamtaufkommens erreicht wird – hier ist offenbar ein Standard gesetzt. Für die VC ist dies aktuell noch nicht der Fall. Ein möglicher Grund sind neben wirtschaftlichen Faktoren auch die Herangehensweisen der Kliniken. In der Regel beginnt eine Klinik mit einem RAS-System. Dieses kann von einer Fachabteilung oder auch interdisziplinär genutzt werden. Die Verfügbarkeit von Operationszeit am RAS-System ist in der Früh- und Lernphase ein zentraler Faktor für die erfolgreiche Etablierung.

Es wird für die breite Akzeptanz und die Etablierung der RAS hilfreich sein, wenn die Pioniere von heute ihre Erfahrungen dokumentieren, analysieren und teilen, damit zukünftige Programme schneller, mit einem höheren Maß an Standardisierung gute Qualität in der Patientenversorgung erbringen können. In den nächsten 10 Jahren wird es vermutlich einen Anstieg an RAS-Systemen in allen chirurgischen Disziplinen geben. Der Wettbewerb wird Möglichkeiten und Limitationen der RAS klarer aufzeigen. Im Zuge der Selbstregulation der Erstattungspraxis des DRG-Systems wird sich mit zunehmendem RAS-Anteil auch das Erstattungsniveau pro Eingriff anpassen. Dieser Effekt wird sich erst ab einem zweistelligen Prozentanteil der RAS an den Prozeduren zeigen – analog zur Prostatektomie (DRG M01B) – und dann zur Anhebung des Relativgewichtes führen.

Von 2014 bis 2018 konnten wir eine Vervierfachung der Eingriffszahlen mit RAS-Systemen registrieren. Besonders im Bereich der VC scheint es einen Bedarf an solchen Systemen zu geben. Hier ist der Einsatz im gleichen Zeitraum sogar um den Faktor 5 gestiegen. Überwiegend waren es onkologisch komplexe bis hoch komplexe Eingriffe, nicht zuletzt auch aufgrund der ausreichend guten Erlössituation.

Das StuDoQ-Register der DGAV hat mit dem Modul Robotik seit 2016 eine Plattform geschaffen, die aktuell zunehmend auch in andere Organmodule integriert wird, um eine umfassendere Dokumentation robotischer Eingriffe zu ermöglichen. Bislang liegen aus diesem Register noch keine Daten vor, die den in dieser Arbeit dargestellten überlegen sind. Die DGAV unterstreicht die Bedeutung der Robotik als dritte technologische Säule der Chirurgie, indem sie zur Begleitung der kurrikularen Ausbildung und Standardisierung die Gründung der damit beauftragten chirurgischen Arbeitsgemeinschaft, CA ROBIN, auf der Mitgliederversammlung 2020 ermöglicht hat. Dies ist eine wichtige Grundlage, um zukünftig RAS-bezogene deutsche Registerforschung zu ermöglichen.

Das medizinische Interesse an dieser jungen Technologie ist weltweit hoch. Etwa 12.000 Publikationen (PubMed [NLM]) im untersuchten Zeitraum und insgesamt über 24.000 Publikationen seit dem Jahr 2000 spiegeln dies wider. Deshalb ist geplant, ein Update dieser Umfrage in 3 Jahren durchzuführen, um weiterhin die Entwicklung der RAS interdisziplinär und dann vielleicht auch mit Systemen anderer Hersteller zu monitoren.

## Fazit für die Praxis

- Die RAS hat sich seit 2014 bis 2018 in Deutschland mit ihrem Eingriffsvolumen interdisziplinär vervierfacht.
- Die Robotik in der VC erlebte seit 2016 eine Verfünffachung des Volumens.
- Die VC ist unter den robotisch arbeitenden Fachabteilungen die Fachrichtung mit dem größten Eingriffsspektrum.
- In der VC sind die kolorektale Chirurgie sowie Eingriffe an Ösophagus und Zwerchfell die Haupteingriffsfelder.
- Die Finanzierung der Technologie erfolgt in 4 von 5 Kliniken über Ankauf der Technologie, aber auch alternative Finanzierungsmodelle wie Miete oder Leasing sind möglich.
- Die Implementierung, das Training und die Etablierung der klinischen Programme durch die Fachabteilungen werden offensichtlich sehr unterschiedlich und bisher nur wenig standardisiert betrieben. Hierfür sollte die DGAV/CA ROBIN zukünftig Vorschläge und Empfehlungen vorlegen.
